# MULTIPLE CHRONIC CONDITIONS AND DISABILITY IN VIETNAMESE OLDER ADULTS IN THE UNITED STATES

**DOI:** 10.1093/geroni/igad104.0169

**Published:** 2023-12-21

**Authors:** Christina Miyawaki, Joshua Garcia, Kim Nguyen, Van Park, Kyriakos Markides

**Affiliations:** University of Houston, Houston, Texas, United States; University of Houston, Houston, Texas, United States; University of Houston, Houston, Texas, United States; University of California, San Francisco, San Francisco, California, United States; University of Texas Medical Branch, Galveston, Texas, United States

## Abstract

Asian Americans recorded the highest life expectancy in the U.S. possibly due to selective migration as 86% of them are immigrants. However, the health of refugees such as older Vietnamese has been understudied. Using data from the Vietnamese Aging and Care Survey (VACS) collected in Houston, Texas, the 2nd-largest Vietnamese-populated metropolitan area in the nation, we assessed their chronic conditions, disability, depressive symptoms, and cognitive impairment, and examined the associations between their chronic conditions and disability by comorbidity clusters. Multivariable logistic regression models were used to answer the research questions. The respondents (N=177) were 76 years old (mean), married (62%), and female (58%). They lived in the U.S. for 24 years (mean) and spoke Vietnamese only (92%) in multi-generation (88%) low-income households (90% ≤$25K). They were in fair/poor health (84%) with 2 ADL/4 IADL disability (mean) and ≥3 chronic conditions (60%). Hypertension (79%) and arthritis (54%) were the most common chronic diseases. Respondents with ≥3 chronic conditions had significantly higher ADL (OR=2.77)/IADL (OR=2.33) disability and cognitive impairment (50%). However, cognitive impairment had the most significant impact on their disability (ADL:2.94-3.75; IADL:5.41-5.79) when combined with most common chronic conditions. Healthcare providers should pay special attention to older Vietnamese’ cognitive impairment due to their high prevalence and negative impact on their disability. Culturally and linguistically tailored healthcare services created by policymakers, healthcare professionals, and local social service agencies are recommended for the well-being of refugees who could not come as selected immigrants but migrated to the U.S. for a better life.

